# Dr. John F. Sanford

**Published:** 1869-02

**Authors:** 


DR. JOHN F. SANFORD.—Our readers will be somewhat surprised to know that Dr. S., for many years a reputable surgeon of this city, has abandoned his infirmary, and is, we understand, about to withdraw from the profession. The Doctor has engaged in patent rights and life insurance. We know nothing of the company he represents, nor the patent lamp burner, of which he is the author, but we trust that in his new sphere of action he will make it more remunerative than professional pursuits. The Doctor is well calculated to fill the new position, and we have no doubt of his financial success.

